# Characteristics of a Bacteriophage, vB_Kox_ZX8, Isolated From Clinical *Klebsiella oxytoca* and Its Therapeutic Effect on Mice Bacteremia

**DOI:** 10.3389/fmicb.2021.763136

**Published:** 2021-12-03

**Authors:** Ping Li, Yangheng Zhang, Fuhua Yan, Xin Zhou

**Affiliations:** ^1^Institute of Comparative Medicine, College of Veterinary Medicine, Yangzhou University, Yangzhou, China; ^2^Jiangsu Co-innovation Center for Prevention and Control of Important Animal Infectious Diseases and Zoonoses, Yangzhou University, Yangzhou, China; ^3^Joint International Research Laboratory of Agriculture and Agri-Product Safety, The Ministry of Education of China, Yangzhou University, Yangzhou, China; ^4^Nanjing Stomatological Hospital, Medical School of Nanjing University, Nanjing, China

**Keywords:** *Klebsiella oxytoca*, biological characteristics, phage vB_Kox_ZX8, phage therapy, bacteremia

## Abstract

*Klebsiella oxytoca* is an important nosocomial and community-acquired opportunistic pathogenic *Klebsiella* and has become the second most prevalent strain in the clinic after *K. pneumoniae*. However, there have been few reports of bacteriophages used for treating *K. oxytoca*. In this study, a novel bacteriophage, vB_Kox_ZX8, which specifically infects *K. oxytoca* AD3, was isolated for the first time from human fecal samples. The biological characteristics of vB_Kox_ZX8 showed an incubation period of 10 min, a burst size of 74 PFU/cell, and a stable *pH* range of 3–11. Genomic bioinformatics studies of vB_Kox_ZX8 showed that it belongs to the genus *Przondovirus*, subfamily *Studiervirinae*, family *Autographiviridae*. The genome of vB_Kox_ZX8 is 39,398 bp in length and contains 46 putative open reading frames encoding functional proteins, such as DNA degradation, packaging, structural, lysin-holin, and hypothetical proteins. We further investigated the efficacy of vB_Kox_ZX8 phage in the treatment of mice with bacteremia caused by *K. oxytoca* infection. The results showed that vB_Kox_ZX8 (5 × 10^9^ PFU/mouse) injected intraperitoneally alone was metabolized rapidly in BALB/c mice, and no significant side effects were observed in the control and treatment groups. Importantly, intraperitoneal injection with a single dose of phage vB_Kox_ZX8 (5 × 10^7^ PFU/mouse) for 1 h post-infection saved 100% of BALB/c mice from bacteremia induced by intraperitoneal challenge with a minimum lethal dose of *K. oxytoca* AD3. However, all negative control mice injected with PBS alone died. Owing to its good safety, narrow host infectivity, high lysis efficiency *in vitro*, and good *in vivo* therapeutic effect, phage vB_Kox_ZX8 has the potential to be an excellent antibacterial agent for clinical *K. oxytoca*-caused infections.

## Introduction

The gram-negative bacterium *Klebsiella*, which exists in the normal flora of the oral cavity, skin, and intestine, is an opportunistic pathogen that can lead to nosocomial infection (Podschun and Ullmann, 1998). *K. oxytoca* is the second most prevalent *Klebsiella* in the clinic after *K. pneumoniae* (Neog et al., 2021). Opportunistic *K. oxytoca* in hospitals mainly infects infants, the elderly, and patients with low immune function, and is the main cause of colitis, endocarditis, sepsis, and urinary and respiratory tract infections (Beaugerie et al., 2003; Ménard et al., 2010; Egger et al., 2017; Tsubouchi et al., 2019; Soto-Hernández et al., 2020; Surani et al., 2020). The prevalence rate of *K. oxytoca* ranges from 2.3 to 24%, accounting for 13–24% of the total nosocomial bacteremia (Watanakunakorn and Jura, 1991; Hansen et al., 1998; Manohar et al., 2017; Ahmad et al., 2018). The high prevalence of *K. oxytoca* warrants extensive attention.

Bacteriophages (phages) are viruses that infect bacteria as their hosts and widely exist in nature. Phages have significant effects on bacteria-phage coevolution and microbial community ecology (Díaz-Muñoz and Koskella, 2014). In addition, there are a large number of phages in the human intestine, which are closely related to health and disease (Manrique et al., 2017; Seo and Kweon, 2019). Recently, owing to the emergence of multidrug-resistant bacteria worldwide, phages have been widely used as substitutes for traditional antibiotics (Kortright et al., 2019). Phage therapy has the advantage of rapid and highly selective bactericidal activity. In addition, phage therapy has a good therapeutic effect on mouse bacteremia, pneumonia, liver abscess, and burn infection caused by *K. pneumoniae* (Hung et al., 2011; Chadha et al., 2017; Kaabi and Musafer, 2019; Anand et al., 2020). However, only a small number of *K. oxytoca* phages have been reported, such as *K. oxytoca* phage ABG-IAUF-1, KLEB010, vB_Klox_2, and PKO111 (Karumidze et al., 2013; Brown et al., 2017; Park et al., 2017; Amiri Fahliyani et al., 2018). Therefore, more *K. oxytoca* phages and their therapeutic effects warrant further investigation. Based on transmission electron microscopy (TEM), there are approximately 10^15^ particles of phages in the human gastrointestinal tract, 10^12^ virus-like particles per gram in fecal samples, and the main families of phages are *Myoviridae, Siphoviridae*, and *Podoviridae* from the order Caudovirales (Dalmasso et al., 2014; Hoyles et al., 2014). With advances in sequencing technology, metagenomic analysis has become the main research method for analyzing intestinal phages. For instance, crAs-like and *Microviridae* phages were found to be the most stable colonizers of the human gut *via* metagenome sequencing (Shkoporov et al., 2019; Koonin and Yutin, 2020). In contrast, the isolation of gut-associated phages was confined to samples derived from sewage from human microbiota samples. Isolation of intestinal phages holds great promise for improving our knowledge of the gut virome, facilitating metagenomic studies, and aiding *in vitro* and *in vivo* studies to investigate the influence of phages on prokaryotic populations in the human gut (Manrique et al., 2017).

In this study, a lytic *K. oxytoca* phage, vB_Kox_ZX8, was isolated from a clinical fecal sample for the first time. The biological characteristics, genomic characteristics, and therapeutic effect of vB_Kox_ZX8 on mice with bacteremia caused by *K. oxytoca* AD3 (AD3) were investigated.

## Materials and Methods

### Animals

Male BABL/C mice (23–25 g) aged 6–8 weeks were purchased and cultured in the experimental animal center of Yangzhou University. All animal experiments were performed in strict accordance with the Regulations for the Administration of Affairs Concerning Experimental Animals approved by the State Council of the People’s Republic of China and the Animal Welfare and Research Ethics Committee at Yangzhou University.

### Host and Culture Conditions

*Klebsiella oxytoca* AD3 was isolated from clinical samples provided by Nanjing Stomatological Hospital in January 2021 and cultured in lysogeny broth (LB) on an orbital shaker at 37°C and 220 rpm. The 16S rRNA gene was amplified using primers 27F and 1492R for identification. K1, K2, K5, K20, and K54 primers were used for serotyping. *RmpA*, *allS*, *ybtA*, *iucB*, *iroNB*, *fimH*, *ureA*, *uge*, *wabG*, and *wcaG* were determined for virulence factors (Supplementary Table 1). Multi-locus sequence typing (MLST) was performed using the primers provided by the Institut Pasteur MLST.^1^ Antibiotic resistance of bacteria was determined using the standard Kirby-Bauer disk diffusion method, and the interpretation of the data was performed using the standardized protocol of the National Committee for Clinical Laboratory Standards and designated as R (resistant), I (intermediate sensitive), and S (sensitive) (Supplementary Table 2).

### Isolation and Purification of Phage vB_Kox_ZX8

Phage vB_Kox_ZX8 was isolated from fecal samples collected from the Nanjing Stomatological Hospital in January 2021. Briefly, 1 g of fecal sample was mixed with 1 mL of PBS, and the solution was mixed well, centrifuged at 8228 × *g* for 5 min, and passed through a 0.22-μm filter. The supernatant filtrate was added to 5 mL of *K. oxytoca* AD3 cultures at log phase (*OD*_600_ = 0.6), and the solution was cultured at 37°C and 220 rpm for 2–4 h. The lysate was centrifuged at 18,514 × *g* for 2 min, and the phage in the supernatant was purified using the double plate method. Briefly, 300 μL of bacterial and 100 μL of 10-fold dilution series of phage were mixed and added to 5 mL of top LB soft agar, followed by pouring onto an LB agar plate and culturing at 37°C overnight. The next day, a single plaque was picked from the plate, inoculated into 5 mL of a log phase host, and cultured at 37°C and 220 rpm for 2–4 h. The cultures were centrifuged at 18,514 × *g* for 2 min, and the lysates were passed through a 0.22-μm filter. The above steps were repeated at least three times until a single phage was obtained.

The phage (MOI = 0.01) was added to 200 mL of log phase host and cultured at 37°C and 220 rpm for 2–4 h. The culture was centrifuged at 8228 × *g* for 10 min, and DNase I and RNase A (1 μg/mL) were added to the lysates and incubated at 37°C for 30 min. NaCl (1 M) was added to the supernatants and incubated in an ice bath for 1 h. After centrifugation at 8228 × *g* for 10 min, 10% (w/v) polyethylene glycol 8000 (PEG 8000) was added to the lysates and precipitated overnight at 4°C. After centrifugation at 18,514 × *g* for 20 min, the precipitate was resuspended in PBS and dissolved completely. An equal volume of chloroform was added to the above solution, and centrifuged at 4629 × *g* for 15 min, followed by transferring the upper water phase to a new centrifuge tube, and the above steps were repeated three times. The collected phages were concentrated in 100 KDa ultrafiltration tubes (Millipore, United States). The phage suspension was then stored at 4°C.

### Biological Characteristics of Phage vB_Kox_ZX8

The morphology of the phage was observed using a transmission electron microscope (TEM) HT7800 (Hitachi, Japan). Before observation, the phage suspension was incubated on carbon grids (200 mesh) for 10 min, stained with 2% phosphotungstic acid for 3 min, and dried for 30 min.

The host spectrum of the isolated phages was determined using the spot-test assay on 25 bacterial strains isolated from patient fecal samples. The antibacterial activity was assayed against 10 *K. oxytoca*, 10 *K. pneumoniae*, 5 *Escherichia coli*, and 5 *Proteus mirabilis* strains (Supplementary Table 3). Briefly, 5 μL of phage (10^8^ PFU/mL) was spotted onto fresh bacterial lawns and incubated at 37°C for 6–8 h. The experiment was repeated three times.

The one-step growth curve of the phage was measured to calculate its incubation period, outbreak period, and platform period. The phage suspension was added to a fresh host culture (10^8^ CFU/mL) at a multiplicity of infection (MOI) of 0.01. The mixture was incubated at 37°C for 10 min and centrifuged at 18,500 × *g* for 1 min to remove the non-absorbed phages. The precipitate was re-suspended in LB broth (time zero) and cultured at 37°C and 220 rpm. Two parallel samples were taken every 10 min and centrifuged at 18,500 × *g* for 1 min. The phage titer in the supernatant was determined using the double agar plate method. Burst size was computed as the ratio of the final count of released phage particles to the initial count of infected bacterial cells during the latent period (Ciacci et al., 2018). The experiment was repeated three times.

Thermal stability test of phage: Phage suspension (300 μL, 10^8^ PFU/mL) was incubated at 4, 40, 50, 60, 70, and 80°C for 20–60 min. The phage titer was determined using the double-layer plate method.

*pH* stability test of phage: Phage suspension (300 μL, 10^8^ PFU/mL) was incubated at 37°C for 60 min under different *pH* values (2–11). The phage titer was determined using the double-layer plate method. The experiment was repeated three times.

The bactericidal effect of phages *in vitro* was evaluated by measuring the effect of phage on the number of bacteria in the culture tube. Different doses of phage vB_Kox_ZX8 (MOI = 10, 1, 0.1, 0.01, and 0.001) were added to log phase *K. oxytoca* AD3 (5 × 10^8^ CFU/mL) and cultured at 37°C and 220 rpm. The *OD*_600_ of the culture was determined every 10 min using a Smart Microplate Reader (Tecan infinite M200 Pro, Switzerland). The experiment was repeated three times.

### DNA Isolation, Genome Sequencing, and Analysis

Phage DNA was extracted using a Virus Genomic DNA/RNA Extraction Kit (Tiangen Biotechnology Co., Ltd., China). Whole-genome sequencing of the phage was completed by Shanghai Bioengineering Co., Ltd. The phage DNA fragments with a length of approximately 500 bp were randomly interrupted by a Covaris ultrasonic crusher (Covaris, United States), and then purified using hieff NGS DNA selection beads (Yeasen Biotechnology Co., Ltd. China). The sequencing library was constructed using the NEB Next Ultra DNA Library Prep Kit for Illumina (NEB, United States), including terminal repair, adaptor ligation, DNA purification, and library amplification. The DNA library was sequenced on the Illumina hiseqpe150 sequencing platform after passing the quality test. The original sequencing data were filtered first and assembled using new blew 3.0 software.

tRNAs were predicted using tRNAscan-SE2.0^2^ (Lowe and Chan, 2016). The virulence factors and drug resistance of phage genome were compared with Virulence Factors of Pathogenic Bacteria^3^ and The Comprehensive Antibiotic Resistance Database.^4^

The open reading frame was annotated using the RAST annotation server web (Aziz et al., 2008). Automatic annotation was manually reviewed using the BLASTp algorithm against RefSeq proteins deposited in the GenBank database. *Trans-*membrane helical domains and signal sequences were analyzed using Phobius (Käll et al., 2007). Visualization of the phage genome was performed using Easyfig (Sullivan et al., 2011). Phylogenetic analysis was performed using the phage terminase large subunit and major capsid protein of the subfamily *Studiervirinae* reported by the International Committee on Taxonomy of Viruses classification. The protein alignments were obtained using ClustalW, and the phylogenetic trees were generated using the maximum likelihood method with a bootstrap of 1000 in MERGA 6.0.

### Safety of Treatment of Mice With Phage vB_Kox_ZX8

The Toxin Eraser Endotoxin Removal Kit (Genscript Biotechnology Co., Ltd., China) was used to remove endotoxins from the phage. The purified phage suspension was quantified using the ToxinSensor Single Test Kit with Standard (Genscript Biotechnology Co., Ltd, China). Endotoxin concentrations below 0.005–0.01 endotoxin units (EU)/PFU were deemed safe for injection in accordance with published data (Gangwar et al., 2021).

Each mouse in the experimental group was intraperitoneally (IP) injected with 100 μL of purified phage vB_Kox_ZX8 (5 × 10^9^ and 5 × 10^7^ PFU), and 100 μL of PBS was injected as a control, with five mice in each group. The weight and survival rates of the mice in each group were observed. The titers of phages in blood and tissues were determined using the double-layer plate method. The levels of tumor necrosis factor-α (TNF-α), interleukin 6 (IL-6), and interleukin 10 (IL-10) in organs were determined using ELISA.

### Mouse Model of Bacteremia

*Klebsiella oxytoca* AD3 bacteria were cultured overnight and then centrifuged at 8228 × *g* for 2 min, followed by washing twice with PBS. Mice (six mice in each group) in the experimental group were IP injected with 100 μL of different doses of bacterial suspension (5 × 10^8^, 10^8^, 5 × 10^7^, 10^7^, 5 × 10^6^, and 10^6^ CFU) to determine the minimum lethal dose (MLD). MLD is the dose of bacteria that could cause all mice to die within 7 days. PBS (100 μL) was injected into the negative control group. The weight and survival rate of the mice in each group were recorded. Each mouse was IP injected with 100 μL of bacteria at the MLD to prepare the bacteremia mouse.

### Phage vB_Kox_ZX8 Treatment Rescues *K. oxytoca* AD3-Infected Mice

Each mouse was IP injected with 100 μL of *K. oxytoca* AD3 at the MLD. After 1 h, different doses of phage vB_Kox_ZX8 (5 × 10^7^, 5 × 10^6^, 5 × 10^5^ PFU) were IP injected into the experimental group, and 100 μL of PBS was injected as a control, with six mice in each group. The weight and survival rates of the mice in each group were recorded. The titers of *K. oxytoca* AD3 and phage vB_Kox_ZX8 in blood and organs were determined using bacterial monoclonal plate culture and double plate experiments, respectively.

After 48 h, the animals were euthanized, and tissues were collected. The organs were fixed with 4% paraformaldehyde for 24 h, dehydrated in alcohol, paraffin-embedded, sectioned, xylene dewaxed, hematoxylin and eosin stained, dehydrated in alcohol, and covered with glass. Pathological changes in the organs of the mice were observed.

### Quantitation of Bacteremia and Phage in Mouse Blood and Tissue

Blood and various organs, including the eyeball, blood, heart, lungs, thymus, right upper liver, spleen, right kidney, and small intestine (2–4 cm downward from the pylorus) were collected immediately after the mice were euthanized. Blood was diluted with a PBS gradient immediately after collection. Each organ, including the small intestine, was homogenized with 1 mL of PBS and then diluted with a PBS gradient. The titers of bacteria and phage in blood and organ homogenates were determined using plate counting and the double plate method, respectively.

### Statistical Analysis

All data were analyzed using GraphPad Prism 6. Statistical analysis of significance was also undertaken using the GraphPad Prism program *via* unpaired *t* test with a *p* < 0.05 considered significant.

## Results

### The Characteristics of *K. oxytoca* AD3 and Phage vB_Kox_ZX8

*Klebsiella oxytoca* AD3 was typed as ST367 and K1 serotype and encodes virulence factor genes *rmpA*, *kfuBC*, *ybtA*, *fimH*, *uge*, *wabG*, and *wcaG*. The strain is sensitive to most antibiotics but resistant to erythromycin, vancomycin, and tobramycin (Supplementary Table 2).

**FIGURE 1:**
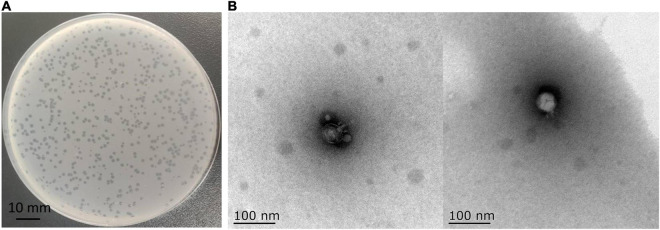
**(A)** Plaques formed by phage vB_Kox_ZX8 on a lawn of the host *K. oxytoca* AD3; **(B)** Transmission electron microscopy of phage vB_Kox_ZX8.

In this study, a phage named vB_Kox_ZX8 was isolated using *K. oxytoca* AD3. The phage formed small circular translucent plaques (diameter < 1 mm) on the lawns of the host (Figure 1A). Examination of phage morphology using TEM analysis showed that phage vB_Kox_ZX8 had an icosahedral head with a dimension of 53 ± 3.0 nm and a very short non-contractile tail (Figure 1B).

A total of 25 clinical isolates were used to evaluate the host range of vB_Kox_ZX8 (Supplementary Table 3). The results showed that phage vB_Kox_ZX8 only had lytic activity specific to *K. oxytoca* AD3. The narrow host spectrum of phages may be due to the limitations of the tested strains or the specificity of phage recognition sites.

### One-Step Growth Curve and Stability Studies of Phage vB_Kox_ZX8

**FIGURE 2:**
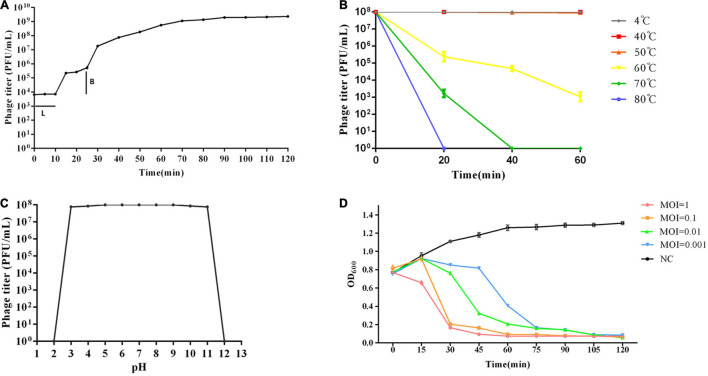
Biological characteristics of phage vB_Kox_ZX8. **(A)** One-step growth curve of phage vB_Kox_ZX8. L: latency period; B: burst size; **(B)** Thermal stability of phage vB_Kox_ZX8; **(C)** *pH* stability of phage vB_Kox_ZX8; **(D)** Killing dynamic of phage vB_Kox_ZX8 against *K. oxytoca* AD3. Viable cell count (*OD*_600_) of cultures infected with vB_Kox_ZX8 at MOI of 1, 0.1, 0.01, and 0.001, and the uninfected control (NC) are shown.

A one-step growth curve was used to analyze the adsorption velocity, latency period, and burst size of the phage (Figure 2A). The results showed that phage vB_Kox_ZX8 had a latency period of 10 min, followed by a rise period of 60 min and a growth plateau of approximately 50 min. The burst size of vB_Kox_ZX8 was computed as 74 phage particles per infected bacterium.

The thermal stability test showed that the phage was relatively stable between 4 and 50°C. After incubation at 60°C for 20, 40, and 60 min, the phage titer (10^8^ PFU/mL) decreased to 2 × 10^5^, 5 × 10^4^, and 10^3^ PFU/mL, respectively. After incubation at 70°C, phage activity decreased to 2 × 10^3^ PFU/mL at 20 min and was completely lost at 40 min (Figure 2B). The *pH* stability test showed that phage activity was very stable at *pH* 3–11, and sharply lost at *pH* = 2 and *pH* = 12 (Figure 2C).

### Killing Dynamic of Phage vB_Kox_ZX8 Against the *K. oxytoca* AD3 Strain

Different doses of phages (MOI = 10, 1, 0.1, 0.01, 0.001, respectively) were used to infect the host *K. oxytoca* AD3 in the log phase, and the *OD*_600_ of the culture was determined using a Smart Microplate Reader to evaluate the killing effect of phages on the host. The results showed that the number of bacteria decreased gradually after the addition of phages, and the rate of decrease was proportional to the number of phages. Even if the MOI of phages was 0.001, the bacteria were almost cleared after 90 min (Figure 2D). This result showed that phage vB_Kox_ZX8 had a good *in vivo* anti-*K. oxytoca* AD3 effect.

### Genome Analysis of Phage vB_Kox_ZX8

The genome of phage vB_Kox_ZX8 (GenBank accession no. MZ424865) is a dsDNA that comprises 39,398 bp with a G+C content of 53.1%. tRNA was not predicted using tRNAscan-SE2.0. Virulence, toxin proteins, and drug-resistance genes were not detected. Whole-genome comparative analysis of phages showed that phage vB_Kox_ZX8 was most closely related to *Klebsiella* phage 2044-307w (95.05%), which is a member of the genus *Przondovirus*, subfamily *Studiervirinae*, family *Autographiviridae*.

**FIGURE 3:**
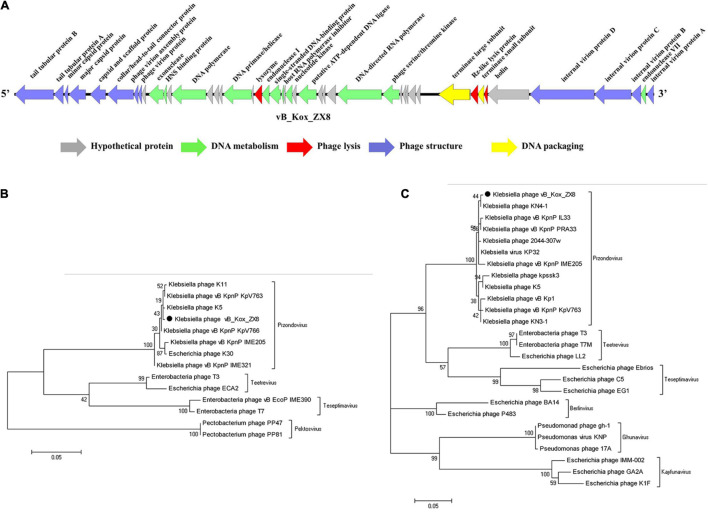
Genome analysis of phage vB_Kox_ZX8. **(A)** Linear genome map of phage vB_Kox_ZX8. Phylogenetic trees of the **(B)** terminase large subunit, and the **(C)** major capsid protein of phage vB_Kox_ZX8.

The genome contains 46 coding sequences or open reading frames (ORFs) (Table 1). The ORFs of phage vB_Kox_ZX8 were divided into five modules: hypothetical protein, DNA metabolism, DNA packaging, phage structure, and phage lysis (Figure 3A). The main functional ORFs of phages are involved in DNA replication and degradation (ORF10, ORF11, ORF15, ORF17, ORF20, ORF21, ORF24, ORF26, ORF45, and ORF35); transcriptional factors (ORF13, ORF22, ORF30, and ORF31); phage capsid and scaffold (ORF3, ORF4, ORF5, and ORF6); phage tail (ORF1, ORF2, ORF7, ORF8, ORF41, ORF42, ORF43, and ORF44), phage assembly (ORF37 and ORF39), and phage lysis (ORF19, ORF38, and ORF40). ATG was proposed as a start codon for 41 ORFs, four ORFs used GTG, and one ORF used TTG. Signal peptides and transmembrane domains were found in seven ORFs: hypothetical protein ORF18, ORF29, ORF34, terminase large subunit ORF37, Rz-like lysis protein ORF38, holin class II ORF40, and internal virion protein B ORF44. The terminal large subunit and internal virion protein B are involved in the assembly of phage DNA. Holin forms pores on the inner membrane, and Rz-like lysis protein assists the lysozyme to enter the outer membrane from the inner membrane and cause complete cell lysis.

**TABLE 1:** Open reading frames (ORFs) of phage vB_Kox_ZX8.

ORFs	Start	Stop	Length (bp)	ORF orientation (+−)	Start codon	Function
ORF1	2416	41	2376	–	ATG	Tail tubular protein B
ORF2	3017	2439	579	–	ATG	Tail tubular protein A
ORF3	3304	3083	222	–	GAG	Minor capsid protein
ORF4	4392	3361	1032	–	ATG	Major capsid protein
ORF5	5626	4667	960	–	ATG	Capsid and scaffold protein
ORF6	7337	5730	1608	–	ATG	Collar/head-to-tail connector protein
ORF7	7621	7361	261	–	ATG	Phage virion assembly protein
ORF8	7844	7623	222	–	ATG	Phage virion protein
ORF9	8092	7847	246	–	ATG	Hypothetical protein
ORF10	9176	8271	906	–	GAG	Exonuclease
ORF11	9382	9173	210	–	ATG	HNS binding protein
ORF12	9666	9379	288	–	ATG	Hypothetical protein
ORF13	11802	9685	2118	–	ATG	DNA polymerase
ORF14	12210	11857	354	–	ATG	Hypothetical protein
ORF15	12594	12283	312	–	ATG	Inhibitor of host toxin/antitoxin system
ORF16	12803	12594	210	–	ATG	Hypothetical protein
ORF17	14605	12875	1731	–	ATG	DNA primase/helicase
ORF18	14724	14602	123	–	ATG	Hypothetical protein
ORF19	15243	14788	456	–	ATG	Lysozyme/N-acetylmuramoyl-L-alanine amidase
ORF20	15695	15246	450	–	ATG	Endonuclease I
ORF21	16390	15695	696	–	ATG	Single-stranded DNA-binding protein
ORF22	16599	16450	150	–	ATG	Host RNA polymerase inhibitor
ORF23	16690	16565	126	–	ATG	Hypothetical protein
ORF24	17108	16677	432	–	ATG	Nucleotide kinase
ORF25	17364	17101	264	–	ATG	Hypothetical protein
ORF26	18597	17482	1116	–	ATG	Putative ATP-dependent DNA ligase
ORF27	18963	18697	267	–	ATG	Hypothetical protein
ORF28	19143	18967	177	–	ATG	Hypothetical protein
ORF29	19790	19227	564	–	ATG	Hypothetical protein
ORF30	22609	19889	2721	–	ATG	DNA-directed RNA polymerase
ORF31	23703	22681	1023	–	ATG	Phage serine/threonine kinase involved in host transcription shutoff
ORF32	23861	23742	120	–	GAG	Hypothetical protein
ORF33	24117	23920	198	–	ATG	Hypothetical protein
ORF34	24241	24095	147	–	ATG	Hypothetical protein
ORF35	24708	24241	468	–	ATG	S-adenosyl-L-methionine hydrolase
ORF36	25001	24735	267	–	ATG	Hypothetical protein
ORF37	28099	26342	1758	–	TTG	Terminase large subunit
ORF38	28542	28096	447	–	ATG	Rz-like lysis protein
ORF39	28896	28639	258	–	ATG	Terminase small subunit
ORF40	29139	28930	210	–	ATG	Holin class II
ORF41	31686	29149	2538	–	ATG	Tail fiber
ORF42	35714	31749	3966	–	ATG	Internal virion protein D
ORF43	37986	35731	2256	–	ATG	Internal virion protein C
ORF44	38576	37986	591	–	ATG	Internal virion protein B
ORF45	38881	38576	306	–	GAG	Endonuclease VII
ORF46	39365	38955	411	–	ATG	Internal virion protein A

Phylogenetic analysis of the phage terminase large subunit and major capsid proteins showed that vB_Kox_ZX8 belonged to the genus *Przondovirus*, subfamily *Studiervirinae*, and family *Autographiviridae* (Figures 3B,C).

### Effect of Treatment With Phage vB_Kox_ZX8 on Mice

**FIGURE 4:**
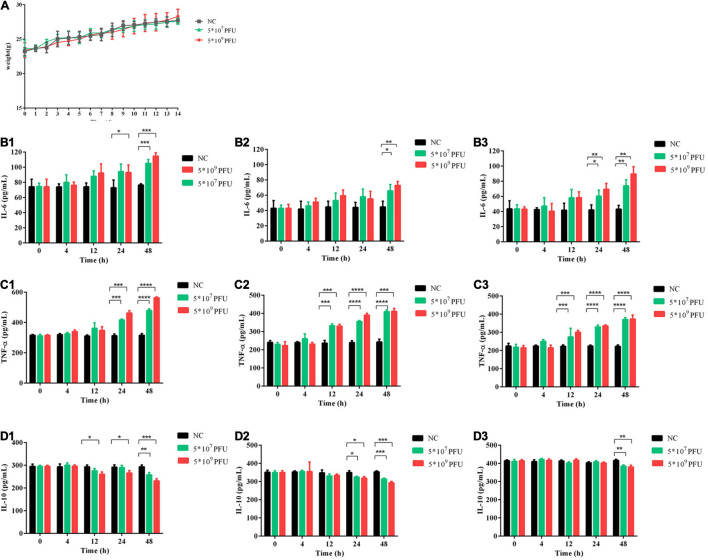
Safety evaluation of phage vB_Kox_ZX8 in mice. **(A)** The weight of mice after injection with phage vB_Kox_ZX8; IL-6 levels in the **(B1)** serum, **(B2)** liver, and **(B3)** spleen of mice after injection with phage vB_Kox_ZX8; TNF-α levels in the **(C1)** serum, **(C2)** liver, and **(C3)** spleen of mice after injection with phage vB_Kox_ZX8; IL-10 levels in the **(D1)** serum, **(D2)** liver, and **(D3)** spleen of mice after injection with phage vB_Kox_ZX8. Each mouse was injected with 5 × 10^7^ PFU and 5 × 10^9^ PFU of phage vB_Kox_ZX8, and the uninjected control (NC).

Compared with the control group, phage vB_Kox_ZX8 had no significant effect on the weight of the mice (Figure 4A). In the inflammatory response, phage caused an increase in pro-inflammatory factors (IL-6 and TNF-α) and a decrease in anti-inflammatory factors (IL-10) (Figures 4B–D). The levels of IL-6, TNF-α, and IL-10 in the serum, liver, and spleen began to change after 12 h or 24 h of phage injection, and the changes caused by the high dose of phage (5 × 10^9^ PFU) were more significant. The changes in inflammatory factors induced by phages showed a gradual upward trend within 48 h. At 48 h after administration of 5 × 10^9^ PFU phage to healthy mice, the IL-6 levels in the serum, liver, and spleen of mice were 1.5, 1.6, and 2.0 times that of the control group, respectively; the levels of TNF-α in the serum, liver, and spleen of mice were 1.8, 1.7, and 1.7 times that of the control group, respectively; the IL-10 content in the serum, liver, and spleen of mice was 0.8, 0.8, and 0.9 times that of the control group, respectively. The fluctuation of IL-6, TNF-α, and IL-10 levels in mice caused by phage were slight, which is not enough to cause obvious adverse reactions.

**FIGURE 5:**
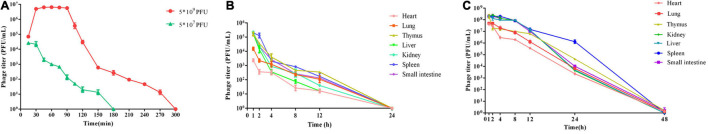
Metabolism in mice after injection with phage vB_Kox_ZX8. **(A)** Phage titer in the blood after mice were injected with 5 × 10^7^ PFU and 5 × 10^9^ PFU of phage vB_Kox_ZX8; phage titer in different organs after phage vB_Kox_ZX8 injection at **(B)** 5 × 10^7^ PFU, **(C)** 5 × 10^9^ PFU.

After intraperitoneal injection of 5 × 10^7^ PFU and 5 × 10^9^ PFU doses of phage vB_Kox_ZX8 to healthy mice, the phages disappeared in the blood after 3 and 5 h, respectively (Figure 5A). In mice injected IP with 5 × 10^9^ PFU, the phage titer in the blood reached the highest (6 × 10^5^ PFU/mL) at 30 min and lasted for 60 min, then dropped rapidly until it was completely undetectable. In mice injected IP with 5 × 10^7^ PFU, the phage titer in the blood reached the highest (2 × 10^4^ PFU/mL) at 15 min and lasted for 30 min, then dropped rapidly until it was completely undetectable. Phage vB_Kox_ZX8 had a higher titer and longer residence time in mouse organs because phages at 5 × 10^7^ PFU and 5 × 10^9^ PFU disappeared in organs within 24 and 48 h, respectively (Figures 5B,C). The titer of vB_Kox_ZX8 in the heart, thymus, lung, liver, spleen, kidney, and small intestine reached the highest levels within 1 h, and then gradually decreased. However, the titers of phages in the liver, spleen, kidney, and small intestine were significantly higher than those in the heart and lung after injection, which may be related to the injection method. At 12 h after 5 × 10^7^ PFU phage injection, the phage titer of the thymus was the highest, followed by the spleen. At 24 h after 5 × 10^9^ PFU phage injection, the phage titer of the spleen was the highest, followed by that of the thymus.

### Mouse Model of Bacteremia

**FIGURE 6:**
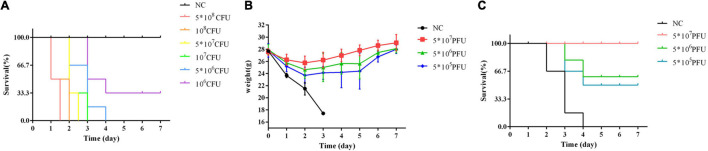
Therapy with phage vB_Kox_ZX8 rescues bacteremia mice. **(A)** The survival rate of mice infected with *K. oxytoca* AD3 and uninfected control (NC); **(B)** The weight and **(C)** Survival rate of bacteremia mice rescued by phage vB_Kox_ZX8 and unrescued control (NC).

After the injection of *K. oxytoca* AD3, the mice were dispirited, and showed inverted hair, shivering, drowsiness, hunched back, weight loss, and death. Injection with a dose of 5 × 10^6^ CFU, which is regarded as the MLD, caused the death of all six mice (Figure 6A).

### Therapy With Single Phage Rescues Bacteremia Mice

The weight of bacteremic mice rescued by phage vB_Kox_ZX8 increased gradually after 2 days (Figure 6B). The survival rate of mice rescued with 5 × 10^7^ PFU of phage reached 100%, and mice rescued with 5 × 10^6^ PFU and 5 × 10^5^ PFU of phage were 66% and 50% of survival rate, respectively (Figure 6C).

**FIGURE 7:**
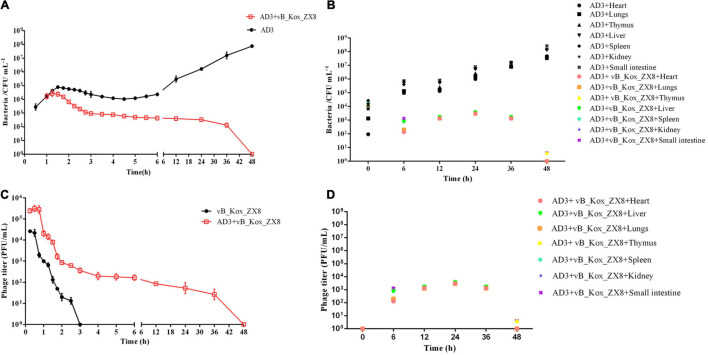
Changes in bacterial and phage titers in bacteremia mice after phage vB_Kox_ZX8 rescue. Bacteria titer in the **(A)** blood and **(B)** different organs of bacteremia mice rescued by phage vB_Kox_ZX8; phage titer in the **(C)** blood and **(D)** different organs of bacteremia mice rescued by phage vB_Kox_ZX8.

Therefore, 5 × 10^7^ PFU phages were selected to rescue bacteremia mice, and the therapeutic effect of the phage was evaluated by measuring the titer of bacteria and phage in mice. The results showed that the bacterial titers in the blood and organs of mice treated with phage decreased gradually and were cleared after 48 h, while the bacterial titers in the blood and organs of mice in the negative control group increased and killed the mice (Figures 7A,B). After the phage entered the body of the mouse, it was first reproduced through *K. oxytoca* AD3, then gradually decreased, and was completely metabolized after 48 h in the blood (Figure 7C). The changes in the number of phages in the organs seem to be related to the bacteria count, and they were cleared after 48 h (Figure 7D). In organs, the bacteria count was decreased by the phages in a short time (<6 h), followed a slight upward trend, and finally cleared after 48 h. Bacteria isolated from the organs and blood were still sensitive to phage vB_Kox_ZX8 at 12, 24, and 36 h post-infection, indicating that bacteria and phages co-exist between 12 and 36 h in mice. However, the phage-resistant strains were isolated after 12 h of coculture *in vitro*. The reason for this phenomenon may be that phages in mice have less selective pressure on bacteria, and a higher phage therapeutic dose should be considered.

**FIGURE 8:**
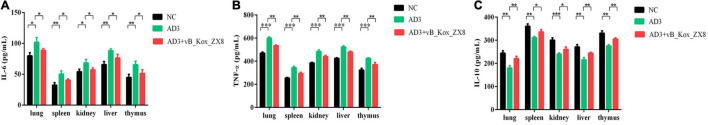
Changes in immune factors in mice after phage vB_Kox_ZX8 rescue. **(A)** IL-6, **(B)** TNF-α, and **(C)** IL-10 levels in mice serum after injection with 5 × 10^6^ CFU of *K. oxytoca* AD3 and 5 × 10^7^ PFU of phage vB_Kox_ZX8, and the uninjected control (NC) are shown.

**FIGURE 9:**
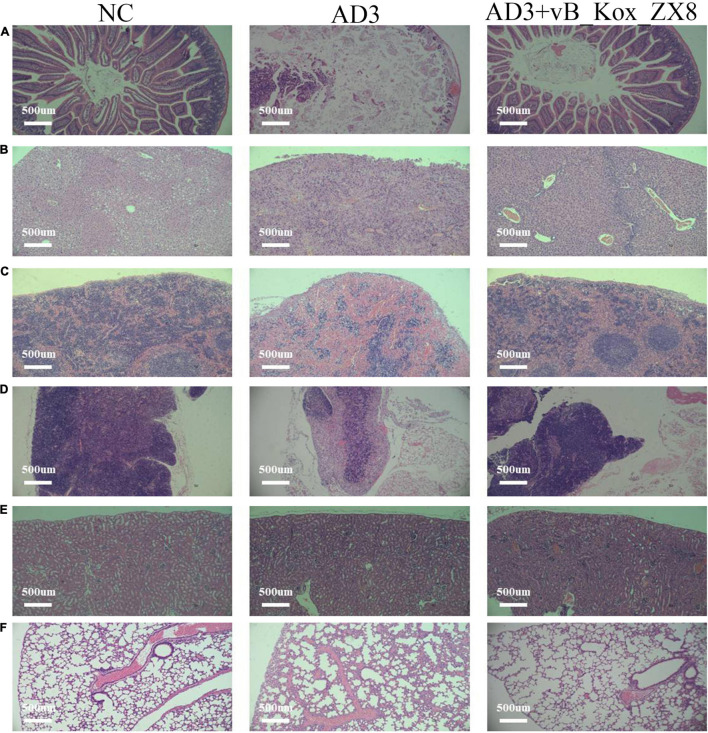
Pathological sections of the **(A)** small intestine, **(B)** liver, **(C)** spleen, **(D)** thymus, **(E)** kidney, and **(F)** lung in mice after 48 h rescued by phage vB_Kox_ZX8. Each mouse was injected with 5 × 10^6^ CFU of *K. oxytoca* AD3 and 5 × 10^7^ PFU of phage vB_Kox_ZX8, and the uninjected control (NC).

After 48 h of infection with *K. oxytoca* AD3, the levels of IL-6 and TNF-α in the thymus, lung, spleen, kidney, and liver of mice increased significantly, while IL-10 decreased significantly, which was effectively alleviated *via* phage therapy (Figures 8A–C). *K. oxytoca* AD3 had the strongest pathogenicity in the small intestine, with obvious pathological changes, hyperemia, cell necrosis, and fuzzy structure (Figure 9A). The number of inflammatory cells in the thymus and spleen decreased significantly and inflammatory cell infiltration was observed in the liver (Figures 9B–D). No obvious pathological changes were observed in the kidneys and lungs (Figures 9E,F). Notably, the pathological changes in various organs in mice rescued by phage showed significant improvement.

## Discussion

The global trend of antibiotic resistance is an urgent problem that needs to be addressed. Based on horizontal gene transfer, some infections caused by pandrug-resistant bacteria cannot be cured with all available antibiotics (Magiorakos et al., 2012). Owing to the high pathogenicity and mortality of multidrug-resistant bacteria, new and effective treatments need to be developed. Phage is a good alternative to antibiotics because of its fast bactericidal effect, high specificity, and low cost. The rapid bactericidal effect of phages has been widely reported in animal models, such as mice, chickens, and cattle. For example, phages are used to treat pneumonia caused by *K. pneumoniae*, enteritis caused by *Salmonella enteritidis* and bovine mastitis caused by *Staphylococcus aureus* (Lim et al., 2012; Anand et al., 2020; Titze and Krömker, 2020). Successful cases of phage therapy in the clinic also show its rapidity and effectiveness, such as phages for prosthetic joint infections caused by *Pseudomonas aeruginosa* or *K. pneumoniae* (Cano et al., 2021; Ferry et al., 2021). At present, phages can be used for treatment only when patients are infected with multidrug-resistant bacteria or those in the antibiotic crisis of incurable infection in clinical practice (Lin et al., 2017). Moreover, some studies have shown that phages are beneficial for reducing the occurrence of antibiotic resistance. For example, the introduction of phages drives bacteria to expel multidrug resistance clusters or decrease antibiotic susceptibilities in antibiotic resistance-related gene expression (Uddin et al., 2019; Majkowska-Skrobek et al., 2021). Phage therapy has great potential applications, but phage resistance and phage pharmacodynamic and pharmacokinetic obstacles remain important challenges (Dąbrowska, 2019; Dąbrowska and Abedon, 2019; Pires et al., 2020).

In this study, *Klebsiella* phage vB_Kox_ZX8 was isolated from human feces, which has a narrow host spectrum. Generally, the tail fiber protein of phages is involved in the adsorption of phages and determines the host range (Bertozzi Silva et al., 2016). BLASTx analysis of phage tail fiber protein showed that two putative conserved domains were detected. The N-terminal of the tail fiber protein (1–154 amino acids) has high homology with T7-like phage tail fiber proteins, such as *Klebsiella* phage K5-4, *Klebsiella* phage SH-KP152226, and *Klebsiella* virus KP32, which have wide host ranges because they encode depolymerase (Hsieh et al., 2017; Pyra et al., 2017; Wu et al., 2019). The C-terminal of the tail fiber protein (amino acids 373–538) had high homology with the lysophospholipase L1-like subgroup of SGNH-hydrolases, which had low homology with other phage proteins. The characteristics of tail fiber proteins suggest that phage vB_Kox_ZX8 may have special recognition sites on the host. Phage vB_Kox_ZX8 has a latent period of less than 10 min, a medium-sized burst of 74 pfu/cell and is stable at *pH* (3–11) and temperature (4–50 °C). In LB medium, the phage with MOI = 1 could reduce the *OD*_600_ of *K. oxytoca* AD3 from 0.8 to 0.1 within 45 min. Among the previously reported *Podoviridae* phages, the incubation period was 10–40 min, the burst size was 120–200 pfu/cell, and *pH* stability was 4–11 (Manohar et al., 2019; Shi et al., 2020; Sofy et al., 2021). In this study, the short incubation period, wide *pH* stability, and special host indicate that phage vB_Kox_ZX8 can be considered as a component of the phage cocktail for treatment.

The current consensus is that phage therapy is safe on the premise that phage preparations are fully purified to ensure low endotoxin levels and remove other bacterial impurities (Speck and Smithyman, 2016; Wienhold et al., 2019). Many studies have reported that phage preparation does not cause changes in inflammatory factors in animals. For example, phage D29 administered *via* the endotracheal route did not cause significant changes in leukocytes, neutrophils, lymphocytes, and TNF-α levels in the lungs of healthy mice 24 h after treatment (Liu et al., 2016); phage BcepIL02 administered *via* IP injection did not cause significant changes in TNF-α levels in the lungs of healthy mice 24 h after treatment (Carmody et al., 2010); and phage Kp_Pokalde_002 administered *via* IP injection did not cause significant changes in TNF-α and IL-6 levels in the plasma of healthy mice 24 h after treatment (Dhungana et al., 2021). In this study, administration of high-dose phage vB_Kox_ZX8 caused slight changes in TNF-α, IL-6, and IL-10 in the serum, liver, and spleen of mice after 24 h of treatment, but did not induce discomfort in mice. This means that the purified phage preparation may contain a small amount of endotoxin and bacterial protein or nucleic acid, which can induce pro-inflammatory reactions in mice. After administration of high-dose PEV31 phage, TNF-α in mice was transiently upregulated at 4 h, and returned to the baseline level after 24 h (Chow et al., 2020). The level of phage preparation-induced inflammation may be temporary and will decrease over time. In addition, it has been reported that some phages can trigger endotoxin-independent inflammatory and anti-inflammatory responses, thereby reducing bacterial clearance to promote phage reproduction (Van Belleghem et al., 2017).

After 5 × 10^9^ PFU of phage vB_Kox_ZX8 was IP injected into healthy mice, the phage titer in the blood was detected within 10 min, maintained at a high level at 30–90 min, gradually decreased, and disappeared after 6 h. In a study of *Podoviridae* phage kpssk3, which has 81% sequence similarity to vB_Kox_ZX8, *Klebsiella* phage kpssk3 showed a similar short residence time in mouse blood. After intraperitoneal injection of 10^8^ PFU phage kpssk3 for 15 min, the blood titer of phages reached 10^5^ PFU/mL, gradually decreasing after maintaining the high titer state for 4 h, and disappeared completely after 8 h (Shi et al., 2021). In other studies of the *Podoviridae* phage, *Klebsiella* phage Kp_Pokalde_002 and *Pseudomonas* phage PEV20 had a longer residence time in mouse blood. The titer of Kp_Pokalde_002 in the blood measured *via* IP injection of 10^8^ PFU reached the maximum at the fourth hour, gradually decreased, and cleared at 48 h (Dhungana et al., 2021). The titer of PEV20 in rat blood measured *via* intravenous injection of 10^8^ PFU reached the maximum within 1 h, gradually decreased, and cleared at 48 h (Lin et al., 2020). In previous reports, active phages were detected in the circulation within the first hour (even less than 5 min) (Bogovazova et al., 1991, 1992; Chhibber et al., 2008). The clearance time of phages in the blood seems to depend on the dose and size of phages, but there is still a knowledge gap between similar phages. In clinical practice, the short life span of phages in the blood is considered an unfavorable factor for phage therapy (Barr, 2017). Some studies have shown that the encapsulation of phages in microparticles and nanoparticles is not only conducive to the storage of phage preparations, but also prolongs the action time of phages *in vivo* (Singla et al., 2016; Malik et al., 2017).

Phages vB_Kox_ZX8 have a longer residence time in the organs than in the blood, and the residence time of phages injected IP at 5 × 10^9^ PFU into the blood (6 h) and organs (48 h) was significantly different. It has been reported that the phage can enter the organ from the blood within a few minutes after administration, and the phage titer in the organ is higher than that in the blood within the first 3 h (Cerveny et al., 2002; Tiwari et al., 2011; Trigo et al., 2013). The acquisition of phages by these organs is usually regarded as a form of phage clearance from the blood, rather than phage distribution. The highest phage titer in mice was lower than the actual injection dose (including vB_Kox_ZX8), indicating that the phage was rapidly captured and neutralized after entering mice; for example, by the mononuclear phagocyte system. The liver and spleen contain many phagocyte precipitates, which are considered to be the main organs for phage clearance from animals and humans, and actively participate in phage neutralization (Lin et al., 2017; Dąbrowska and Abedon, 2019). During the metabolism of phage vB_Kox_ZX8, the spleen accumulated more phages than the liver, which may be because the spleen is more effective in filtering phage vB_Kox_ZX8. A circulating study of intravenous T4 phage in a mouse model showed that the liver has a higher accumulation concentration and faster elimination time for phage, which proves that Kupffer cells in the liver are very effective for the rapid removal of phage particles (Inchley, 1969; Kaźmierczak et al., 2021). The thymus also has high aggregation of phage vB_Kox_ZX8, which has rarely been mentioned in previous studies. The thymus, an important immune organ, may play an important role in phage clearance. According to the literature, some phages can enter most organs in the body including the bones, bladder, skin, salivary glands, and brain, from the blood (Nishikawa et al., 2008; Pouillot et al., 2012; Dąbrowska and Abedon, 2019). The high titer of phage vB_Kox_ZX8 in the small intestine indicates that vB_Kox_ZX8 can easily cross the intestinal barrier and enter the intestine from the blood. Reverse osmosis of phages from the blood to the gastrointestinal tract does not exist in all phages. In previous reports, phages were detected in the feces of calves and mice, the intestines of mice, rabbits, and chickens, and the stomachs of mice (Dąbrowska, 2019).

Phages administered *via* the IP route have the characteristics of higher dose delivery, earlier delivery time, and longer maintenance time, which is correlated with more effective protection of experimental animals from lethal septicemia (McVay et al., 2007). In mice bacteremia induced with *K. oxytoca* AD3, phage vB_Kox_ZX8 showed good therapeutic potential *via* IP injection. The bacterial titers in the blood and organs of mice in the phage-treated group decreased gradually and were cleared after 48 h. The survival rate of the treated group was 100%, while the untreated mice all died, which undoubtedly proved to have a good therapeutic effect. Some studies have shown that a single phage preparation can have a good therapeutic effect in the treatment of mouse bacteremia (Wang et al., 2006, 2018; Vinodkumar et al., 2008; Hung et al., 2011; Alvi et al., 2020). Some of these phages have better therapeutic effects than antibiotics (Sunagar et al., 2010). Recent studies have demonstrated that the combination of phage and antibiotics is more effective than monotherapy in the treatment of bacterial infections (Oechslin et al., 2017; Wang et al., 2021). In the synergistic therapy of phages and antibiotics, phage selection pressure can make bacteria sensitive to antibiotics (Segall et al., 2019). The determination of the type and dose of antibiotics, optimization of combination therapy, and prevention of side effects are very important. Generally, a single phage can effectively reduce the number of bacteria in a short time; however, it is easy to produce phage-resistant bacteria in the later stages of treatment (Hung et al., 2011; Hesse et al., 2021). Phage cocktail has a wider bactericidal range and a lower probability of phage resistant strains, and seems to better protect mice from death caused by bacteremia (Forti et al., 2018; Kaabi and Musafer, 2019). However, the competitive interference between different phages may affect the effectiveness of the phage mixture; therefore, it is necessary to confirm the effectiveness of the phage mixture on a single phage (Geng et al., 2020).

Although most of the phages that have presented good therapeutic effects were isolated from sewage, some lytic phages isolated from the intestine showed high bactericidal ability. *Myoviridae* phage ΦAPCEc01, ΦAPCEc02, and *Siphoviridae* ΦAPCEc03 were isolated from human feces samples, which can inhibit the growth of *E. coli* and reduce the formation of biofilm (Dalmasso et al., 2016). The *Siphoviridae* phage KLPN1 was isolated from a cecal effluent sample, which can lyse *K. pneumoniae* and has depolymerase activity (Hoyles et al., 2015). The *Podovirida*e *Proteus* phage PM16 was isolated from human feces and has characteristics of high stability, a short latency period, large burst size, and low phage resistance (Morozova et al., 2016).

In summary, a novel phage named vB_Kox_ZX8 that specifically infects *K. oxytoca* AD3 was isolated, and this is the first report of *K. oxytoca* phage obtained from a clinical fecal sample. The biological characteristics and rescue experiments in bacteremic mice showed that phage vB_Kox_ZX8 has the potential to be an excellent reagent for infection caused by *K. oxytoca* in the clinic.

## Data Availability Statement

The datasets presented in this study can be found in online repositories. The names of the repository/repositories and accession number(s) can be found in the article/Supplementary Material.

## Ethics Statement

The studies involving human participants were reviewed and approved by Nanjing Stomatological Hospital Medical School of Nanjing University. Written informed consent for participation was not required for this study in accordance with the national legislation and the institutional requirements. The animal study was reviewed and approved by Experimental Animal Ethics Committee of Yangzhou University.

## Author Contributions

XZ and FY supervised the project, analyzed the data, and revised the manuscript. PL and YZ performed the experiments, drew the figures, and wrote the draft manuscript. All authors contributed to the final version of this manuscript.

## Conflict of Interest

The authors declare that the research was conducted in the absence of any commercial or financial relationships that could be construed as a potential conflict of interest.

## Publisher’s Note

All claims expressed in this article are solely those of the authors and do not necessarily represent those of their affiliated organizations, or those of the publisher, the editors and the reviewers. Any product that may be evaluated in this article, or claim that may be made by its manufacturer, is not guaranteed or endorsed by the publisher.

## Footnotes

1
https://bigsdb.pasteur.fr/klebsiella/klebsiella.html

2
http://lowelab.ucsc.edu/tRNAscan-SE/

3
http://www.mgc.ac.cn/VFs/main.htm

4
https://card.mcmaster.ca/
