# Supplementary material for: Characteristics of a Bacteriophage, vB_Kox_ZX8, Isolated From Clinical Klebsiella oxytoca and Its Therapeutic Effect on Mice Bacteremia
Source: Front Microbiol. 2021 Dec 3;12:763136. doi: 10.3389/fmicb.2021.763136 (PMC8678519; doi:10.3389/fmicb.2021.763136)
Supplement: Supplementary file 1 [file Data_Sheet_1.pdf]

Supplementary Material

Characteristics of a bacteriophage vB_Kox_ZX8 isolated from clinic *Klebsiella oxytoca* and its therapeutic effect on mice bacteremia

Ping Li[1,2,3,†], Yangheng Zhang[4,†], Fuhua Yan[4,*], Xin Zhou[1,2,3,*]

[1]Institute of Comparative Medicine, College of Veterinary Medicine, Yangzhou University, Yangzhou 225009, China

[2]Jiangsu Co-innovation Center for Prevention and Control of Important Animal Infectious Diseases and Zoonoses, Yangzhou University, Yangzhou 225009, China

[3]Joint International Research Laboratory of Agriculture and Agri-Product Safety, the Ministry of Education of China, Yangzhou University, Yangzhou 225009, China

[4]Nanjing Stomatological Hospital, Medical School of Nanjing University, Nanjing 210008, China

*** Correspondence:**
Corresponding Author: Xin Zhou, zhou_xin@126.com; or Fuhua Yan, yanfh@nju.edu.cn

[†]These authors contributed equally to this work.

Table 1 Primers used in this study.

**Gene**	**Primer**	**Sequence**
16S rRNA	27F	AGAGTTTGATCCTGGCTCAG
	1492R	GGTTACCTTGTTACGACTT
magA_K1	K1F	GGTGCTCTTTACATCATTGC
	K1R	GCAATGGCCATTTGCGTTAG
wzy_K2	K2F	GACCCGATATTCATACTTGACAGAG

	K2R	CCTGAAGTAAAATCGTAAATAGATGGC
wzx_K5	K5F	TGGTAGTGATGCTCGCGA
	K5R	CCTGAACCCACCCCAATC
wzy_K20	K20F	CGGTGCTACAGTGCATCATT
	K20R	GTTATACGATGCTCAGTCGC
wzx_K54	K54F	CATTAGCTCAGTGGTTGGCT
	K54R	GCTTGACAAACACCATAGCAG
wzy_K57	K57F	CTCAGGGCTAGAAGTGTCAT
	K57R	CACTAACCCAGAAAGTCGAG

Table 2 Antibiotic resistance of *Klebsiella oxytoca* AD3

**Antibiotic**	Piperacillin	Ampicillin	Ceftazidime	Cefuroxime	Cefoxitin
**Drug resistance**	S	I	S	S	S
**Antibiotic**	Cefatriaxone	Cefazolin	Cefotaxime	Imipenem	Aztreonam
**Drug resistance**	S	S	I	S	S
**Antibiotic**	Gentamicin	Levofloxacin	Ciprofloxacin	Trimethoprim	Nitrofurantoin
**Drug resistance**	S	S	S	S	I
**Antibiotic**	Azithromycin	Erythromycin	Sulfamethoxazole	Vancomycin	Tobramycin

Table 3 Host Range of phage vB_Kox_ZX8

	**Bacteria**	**lytic**
Klebsiella oxytoca	AD3	+
	A1	−
	A2	−
	A4	−
	A5	−
	A7	−
	A23	−
	A24	−
	G3	−
	N2	−
Klebsiella pneumoniae	O4	−
	111-2	−
	602-2	−
	AE2	−
	Z1	−
Escherichia coli	AD4	−
	AE1	−
	AG4	−
	X5	−

	W5	−
Proteus mirabilis	AE5	−
	AF1	−
	AA3	−
	V3	−
	W3	−

+: Phage can form plaque ; −: Phage can’t form plaque.
